# Evidence of genetic heterogeneity in Alberta Hutterites with Usher syndrome type I

**Published:** 2012-05-31

**Authors:** Qi Zhou, Chaeli Lenger, Richard Smith, William J Kimberling, Ming Ye, Ordan Lehmann, Ian MacDonald

**Affiliations:** 1 Department of Ophthalmology, Peking Union Medical College, Beijing, China; 2 Genetics Center, Boys Town National Research Hospital, Omaha, NB; 3 Department of Ophthalmology, University of Alberta, Edmonton, Canada; 4 Department of Medical Genetics, University of Alberta, Edmonton, Canada

## Abstract

**Purpose:**

To identify the genetic defect in a Hutterite population from northern Alberta with Usher syndrome type I.

**Methods:**

Complete ophthalmic examinations were conducted on two boys and two girls from two related Hutterite families diagnosed with Usher syndrome type I. DNA from patients and their parents was first evaluated for a mutation in exon 10 of the protocadherin-related 15 (*PCDH15*) gene (c.1471delG), previously reported in southern Alberta Hutterite patients with Usher syndrome (USH1F). Single nucleotide polymorphic linkage analysis was then used to confirm another locus, and DNA was analyzed with the Usher Chip v4.0 platform.

**Results:**

Severe hearing impairment, unintelligible speech, and retinitis pigmentosa with varying degrees of visual acuity and visual field loss established a clinical diagnosis of Usher syndrome type I. The patients did not carry the exon 10 mutation in the *PCDH15* gene; however, with microarray analysis, a previously reported mutation (c.52C>T; p.Q18X) in the myosin VIIA (*MYO7A*) gene was found in the homozygous state in the affected siblings.

**Conclusions:**

The finding of a *MYO7A* mutation in two related Hutterite families from northern Alberta provides evidence of genetic heterogeneity in Hutterites affected by Usher syndrome type I.

## Introduction

Usher syndrome (USH) is an autosomal recessive disorder, characterized by bilateral hearing loss and retinitis pigmentosa (RP). As the most frequent cause of deafblindness, the clinical features of USH are heterogeneous along with the underlying genetics. Clinical examination defines three types: Usher syndrome type I (USH1) with severe to profound congenital hearing impairment, vestibular dysfunction, and retinal degeneration beginning in childhood; type II (USH2) with moderate to severe hearing impairment, normal vestibular function, and later onset retinal degeneration [1]; and type III (USH3) with progressive hearing loss and variable age of onset of retinal degeneration [2]. Some cases are not easily classifiable under these present categories and could be categorized as atypical USH syndrome [3].

The Hutterites are a genetically isolated population that has lived on the North American prairies since the late 1800s [4]. Currently, most Canadian Hutterites live in colonies formed in Alberta, Saskatchewan, and Manitoba in 1918. Since the three groups were established in North America (the Schmiedeleut, Dariusleut, and Lehrerleut), the leuts (groups) have maintained separate identities with the majority of marriages occurring between individuals from the same leut. Lowry and colleagues [5] hypothesized that the lifestyle and nutrition of Hutterite communities might reduce the frequency of congenital anomalies, while consanguinity might serve to increase their frequency. Because of intermarriage within the Hutterite population, rare alleles causing autosomal recessive conditions were likely introduced into the population by one or, at most, a few ancestors. A larger number of monogenic disorders and fewer multifactorial, congenital anomalies are found in the Hutterite population [5].

A mutation in exon 10 of the protocadherin-related 15 (*PCDH15*) gene (c.1471delG) was previously reported in the Hutterite population of southern Alberta [6]. Our colleagues from southern Alberta were not aware of other individuals with Usher syndrome in the province (R. Brian Lowry, Alberta Children's Hospital, Calgary, Alberta, Canada, January 2012). As the Hutterite populations are genetically isolated, we hypothesized a founder effect, that the same mutation would be carried by all Hutterites. Here, we describe two Hutterite families from northern Alberta with Usher syndrome type I who did not carry a mutation in exon 10 of *PCDH15* but did carry a mutation in exon 3 of the myosin VIIA (*MYO7*A) gene, providing evidence of genetic heterogeneity in Alberta Hutterites with Usher syndrome type I.

## Methods

This study was conducted under the tenets of the Declaration of Helsinki and was approved by the Health Research Ethics Board of the University of Alberta. Informed written consent was obtained from the subjects.

### Family data

Patients, two boys and two girls, from two related Hutterite families, were referred to the Department of Ophthalmology, University of Alberta, Canada. A detailed medical history was obtained. Ocular examinations included the measurement of visual acuity, retinoscopy, funduscopy with photographic documentation, and Goldmann visual field examination. DNA was prepared, according to a standard protocol, from peripheral blood samples taken from the parents and all the siblings of the two families. Pure tone audiometry results for individual II:4 at the age 6 and 7 were obtained from the referring physician.

### Mutation analysis

Fluorescently labeled primers were used to amplify exon 10 of *PCDH15*; the molecular size of the amplicon was determined with BI 3100 DNA sequencing and GeneMapper 4.0 software (Carlsbad, CA). Fluorescent microsatellite marker genotyping is accurate down to the base pair level. Individuals carrying a deletion in exon 10 would have an amplicon of decreased size compared to wild-type and all amplicons were sequenced for additional confirmation. Single nucleotide polymorphic (SNP) linkage analysis was first used to confirm another presumed locus for the disorder. DNA was then further analyzed using the Usher Chip v4.0 platform [7]. A pathologic mutation on the array (c.52C>T; p.Q18X) was confirmed to be present with standard Sanger sequencing of DNA from the patients and their parents.

## Results

### Clinical findings

**Figure 1:**
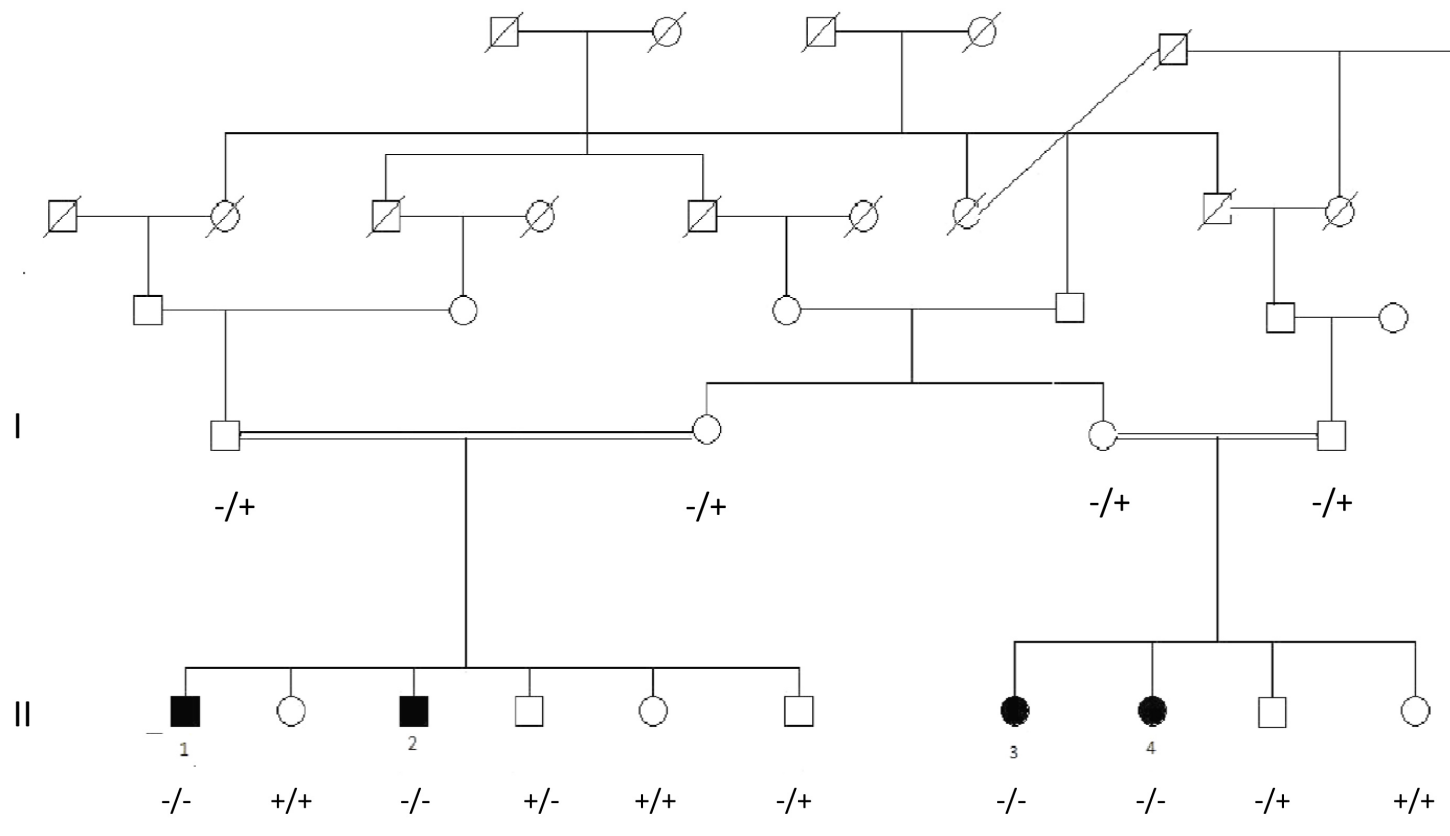
Pedigree of Hutterite families. *MYO7A* mutation c.57C>T (-), normal allele (+).

**Figure 2:**
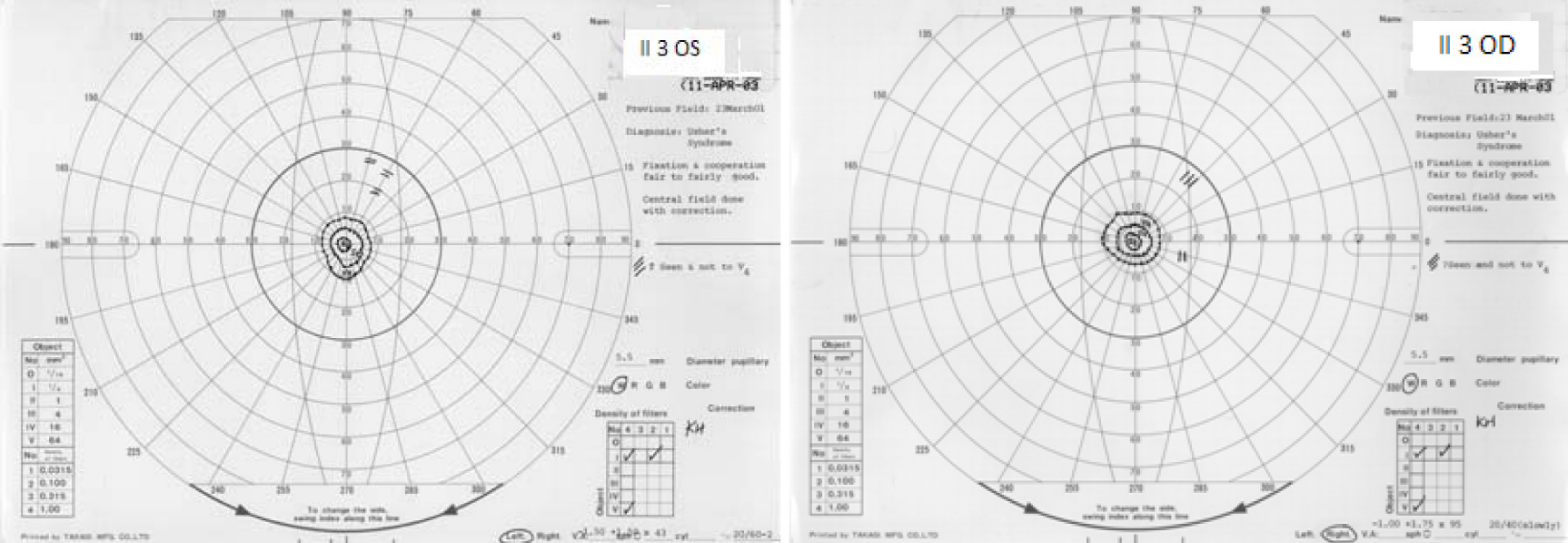
Visual field of patient II:3, age 13. Constricted visual field, central 20 degrees preserved (OU).

**Figure 3:**
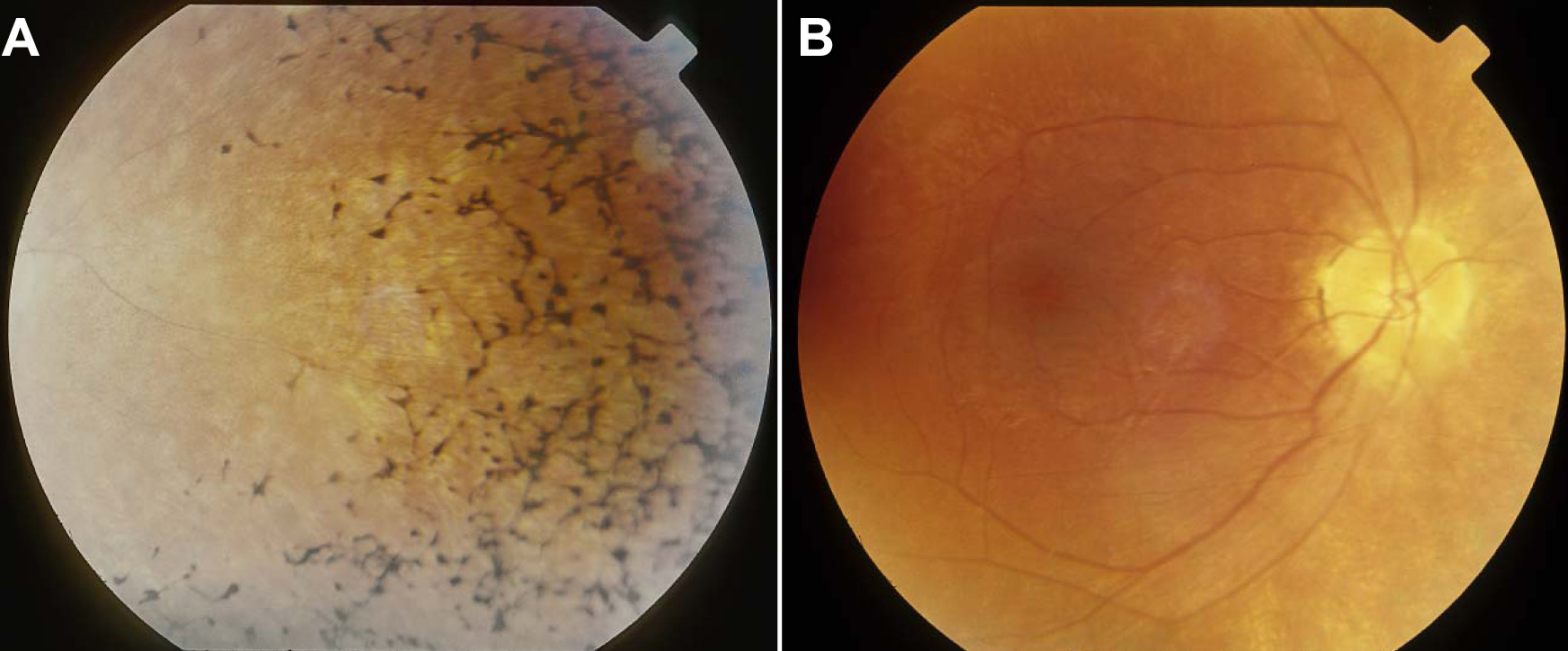
Fundus photographs, OD, of patient II:2 at the age of 10. **A**: Bone spicules in nasal periphery. **B**: Pale optic nerve, vessel attenuation, and normal macula.

Two boys and two girls from two related Hutterite families were diagnosed with Usher syndrome (Figure 1). All four patients had prelingual, bilateral sensorineural hearing loss, and delayed development of walking in early childhood. The pure tone audiometry results from individual II:4 showed hearing thresholds of 25, 60, 75, 75, and 80 dB at 0.25, 0.5, 1, 2, and 4 kHz, respectively, in the right ear, and 35, 65, 75, 55, and 65 dB in the left ear. There was no change in the thresholds one year later. The patients did not develop intelligible speech, and hearing aids were not effective. At the time of referral, all showed evidence of RP. Individuals II:1 and II:2 showed bilateral nasal loss of the visual field, and bone spicules in the nasal fundus at the age of 18 and 10, respectively. Subject II:3 showed bilateral bone spicules in the fundus, and only 20 degrees of visual field was preserved in both eyes by age 13. Subject II:4 showed bilateral nasal loss of the visual field and bone spicules in both eyes at age 9. The ocular findings of the Hutterite patients from northern Alberta are listed in Table 1. Characteristic Goldmann visual fields and fundus photographs are shown in Figure 2 and Figure 3, respectively. The patients’ parents all had normal fundus examinations and normal visual fields. While asymptomatic, no formal tests of balance and hearing were obtained.

**Table 1:** Clinical features of patients with Usher syndrome type I

**Patient**	**Age**	**Vision**	**Refraction**	**Visual field**	**Fundus**
II-1	18	OD:20/25	Plano	Nasal loss	Optic nerve head drusen
		OS:20/25	Plano	Nasal loss	Optic nerve head drusen
II-2	10	OD:20/40	−4.50+0.50×100	Nasal loss	See Figure 3
		OS:20/50	−4.50+0.50×100	Nasal loss	See Figure 3
II-3	13	OD:20/25	−1.00+1.75×95	See Figure 2	Bone spicules
		OS:20/50	−1.50+1.50×43	See Figure 2	Bone spicules
II-4	9	OD:20/50	−0.50+2.00×80	Nasal loss	Bone spicules
		OS:20/200	−0.50+1.75×83	Nasal loss	Bone spicules

### Mutation analysis

No difference in the size of the amplicon of exon 10 of *PCDH15* was observed in the affected individuals compared to unaffected relatives or wild-type controls. In addition, direct sequencing of this amplicon failed to reveal a mutation in any of the four siblings (data not shown). Further, SNP linkage analysis demonstrated linkage to the 11q region, the locus for USH1B. With microarray analysis [7], a point mutation (c.52C>T; p.Q18X) was revealed in exon 3 of the *MYO7A* gene (NM_000260.3; NP_000251.3) segregating in both families. All affected siblings were homozygous for the mutation, and all four parents were heterozygous (Figure 1).

## Discussion

Usher syndrome is the most common cause of bilateral hearing loss and RP, accounting for more than 50% of individuals who are both deaf and blind [8,9], about 18% of RP cases [10], and 5% to 9% of all cases of childhood deafness [11,12]. The prevalence of Usher syndrome varies from 3.2 to 6.2/100,000 depending on the study [8,10,13-15]. The diagnosis is made primarily based on the clinical findings. While auditory and vestibular functions are the distinguishing features of the three different types, RP is the main ophthalmic manifestation shared by all three. Usher patients experience progressive photoreceptor degeneration (RP), which leads to loss of peripheral vision. Clinical symptoms may vary and include night blindness (nyctalopia) with elevated dark adaptation thresholds, abnormal electroretinographic responses, visual field constriction, abnormal retinal pigmentation including peripheral bone spicules, arterial narrowing, and optic nerve pallor, and a predisposition to myopia and posterior subcapsular cataracts [16]. Alteration in the morphogenesis and stability of stereocilia results in sensorineural hearing loss and may also cause balance defects [17].

Usher syndrome type I is the most severe form of this disease. USH1 patients suffer from severe to profound prelingual and bilateral sensorineural hearing loss. These individuals are either born completely deaf or experience hearing impairment within the first year of life and usually do not develop speech. Most, but not all, USH1 patients exhibit severe dysfunction of the vestibular system from birth [3]. Children may manifest a delay in gross motor development; they will sit independently and walk significantly later than normal. The onset of RP in USH1 occurs before puberty and leads to visual field constriction and total blindness afterward. In our Hutterite families, none of the patients developed intelligible speech, indicating prelingual, possibly congenital hearing loss. They all had signs of RP at an early age (in their teens). The history of delayed development of walking was also a clue to vestibular dysfunction and allowed us to assign the diagnosis of USH1 in these families; however, the audiometric results from one patient showed lower hearing thresholds than reported in the literature for USH1. Pakarinen et al. [18] found mean hearing thresholds of about 90, 100, 105, and 110 dB at 0.25, 0.5, 1, and 2 kHz, respectively, in 79 patients with Usher syndrome type I. Wagenaar et al. [19] found minimum hearing thresholds of 80, 95, 120, and 120 dB at 0.25, 0.5, 1, and 2 kHz, respectively, in patients with type I. The reported pure tone average in patients with type II is between 40 and 90 dB [18,20]. The hearing pattern in case II:4 from our families was similar to type II; however, although she began to wear hearing aids at the age of 2, she did not develop intelligible speech, and her hearing remained relatively stable. These findings still confirmed the clinical diagnosis of USH1. There may be genetic modifiers in this Hutterite family that result in the observed differences in hearing. Further study is necessary with more comprehensive evaluations of hearing and vestibular function.

To date, there are five known USH1 genes: *MYO7A* (USH1B), cadherin-23 (*CDH23*; USH1D), *PCDH15* (USH1F), Usher syndrome type 1C (*USH1C*; USH1C), and *SANS* (USH1G) [21]. The myosin VIIa gene (*MYO7A*) was the first identified USH gene [22], and at least 29% to 50% of the known USH1 cases in different populations are caused by mutations in *MYO7A* [23-26]. Mutations in the *MYO7A* gene are also associated with an autosomal recessive form of non-syndromic hearing loss known as DFNB2, and with an autosomal dominant form of hearing loss designated DFNA11. The *MYO7A* gene encodes an unconventional myosin. Myosins are motor molecules with highly divergent tails that move along actin filaments. This movement is presumed to enable them to transport cargo [22]. In the families in this study, a point mutation in the *MYO7A* gene (c.52C>T; Q18X) was carried in the homozygous state in all affected individuals. The mutation creates a stop mutation and truncates the protein product. This could explain the severe phenotype in all four patients and the lack of apparent phenotypic variability. Other mutations in *MYO7A* have been reported to cause a less severe phenotype with significant intrafamilial variability of the trait, such as the c.1935G>A mutation that alters the splicing of exon 16, with exclusion [27].

In summary, we searched for a deletion in exon 10 of the *PCDH15* gene, previously identified in the Hutterites; however, none was found in two related families from northern Alberta. The finding of mutation in *MYO7A* in these families provides evidence of genetic heterogeneity in Hutterites affected by Usher syndrome type I.
